# Effect of individual allergen sensitization on omalizumab treatment outcomes in patients with severe allergic asthma determined using data from the Czech Anti-IgE Registry

**DOI:** 10.1186/s13223-020-00479-1

**Published:** 2020-09-15

**Authors:** Petr Vaník, Jakub Novosad, Olga Kirchnerová, Irena Krčmová, Milan Teřl, Czech Anti-IgE Registry collaborators

**Affiliations:** 1 Department of Respiratory Diseases, B. Němcové 54, Hospital České Budějovice, a.s., Faculty of Medicine in Pilsen, Charles University, 37001 Prague, Czech Republic; 2 Institute of Clinical Immunology and Allergy, University Hospital and Faculty of Medicine, Hradec Kralove, Czech Republic; 3 Department of Pneumology and Phthisiology, University Hospital and Faculty of Medicine in Pilsen, Charles University, Prague, Czech Republic

**Keywords:** Omalizumab, Severe allergic asthma, Allergic sensitization

## Abstract

**Background:**

Omalizumab is an efficient drug for patients with uncontrolled severe allergic asthma (SAA). However, little is known about the differences in omalizumab treatment outcomes among patients with different types of atopic sensitization. Here, we assessed the effect of sensitization to individual allergens or their combinations on the outcomes of anti-IgE therapy in patients with SAA.

**Methods:**

We performed a post hoc analysis of data of subgroups of patients enrolled in the Czech Anti-IgE Registry (CAR). The patients were evaluated at baseline and 16 weeks and 12 months after omalizumab treatment initiation. We analyzed the dependence of primary treatment outcomes [global evaluation of treatment effectiveness (GETE) after 16 weeks of treatment, a reduction in severe exacerbation rate (ER), and an improvement in the asthma control test (ACT) result during 12 months of treatment] and secondary outcomes [a reduction in systemic corticosteroid (SCS) use, an improvement in lung functions, and a fraction of exhaled nitric oxide] of patients with SAA treated with omalizumab for 12 months on sensitization to different perennial aeroallergens. We assessed sensitization to house dust mites, molds, and pets at baseline using skin prick tests and/or specific IgE measurement (semiquantitative evaluation). We compared polysensitized patients (sensitized to all tested allergens) with monosensitized (single positivity) or partially polysensitized patients (combined positivity but not to all allergens).

**Results:**

We enrolled 279 patients (58.3% women, mean age 52.9 years). Omalizumab treatment presented an 82.8% response rate (according to GETE). It significantly reduced severe asthma exacerbations and SCS use, and improved the ACT result in 161 responders. We identified a subgroup of responders with distinct sensitization patterns (polysensitization to all tested perennial allergens) with higher odds of being responders (OR = 2.217, p = 0.02) and lower tendency to improve ACT result (OR 0.398, p = 0.023) and reduce ER (OR 0.431, p = 0.034) than non-polysensitized patients.

**Conclusions:**

The clinical benefit of sensitization for patients with SAA receiving omalizumab may be particularly dependent on sensitization pattern. Polysensitized patients showed a higher tendency to be responders (GETE), but a lower tendency to improve the ACT result and reduce ER than non-polysensitized patients.

## Background

The global prevalence of asthma is still uncertain [1, 2], and some experts suggest a prevalence of 7–8% (i.e., 700 000–800 000 people) in the Czech Republic (CR). In this particular asthma population, probably more than 80% of patients have eosinophilic (or type 2-high) and over 70% have eosinophilic allergic (or Th2- high) asthma endotype [3–7].

Patients with severe asthma [8] (or severe refractory asthma) account for approximately 2.1% of the whole asthmatic population in the CR [3]. Currently, the Global Initiative for Asthma (GINA) recommends targeted biological therapy before the administration of systemic corticosteroids (SCSs) in step 5 of the integrated treatment strategy, wherever possible. The broad clinical effects of omalizumab have been extensively documented in controlled studies [9, 10] and real-world studies [11–15].

However, little is known about the differences in treatment outcomes associated with a particular sensitization type or combinations of aeroallergens. In general, allergic reactivity to perennial allergens (such as house dust mites, molds, dogs, and cats) must be proved to initiate an anti-IgE monoclonal antibody omalizumab treatment [16]. Nonetheless, a previous study has indicated that sensitization to seasonal allergens can lead to a comparable treatment response [17].

In this study, we aimed to evaluate the potential differences in treatment outcomes among patients with severe allergic asthma (SAA) according to the sensitization pattern to different aeroallergens and their combinations. All patients were treated in accordance with local and international guidelines [7, 18, 19] using high-dose inhaled corticosteroids with long-acting β2-agonists or an alternative controller (leukotriene receptor antagonists or theophylline) with an add-on anti-IgE therapy with omalizumab. We used data from the Czech anti-IgE Registry (CAR).

We assessed the following treatment outcomes: (1) responding (according to the global evaluation of treatment effectiveness [GETE] at 16 weeks after treatment initiation), not responding, or withdrawing treatment; (2) a reduction in severe exacerbation rate (ER) after 12 months of treatment in responders compared with that in non-responders and patients who withdrew omalizumab treatment; and (3) an improvement in the asthma control test (ACT) result after 12 months of treatment (in all three study arms) in patients with SAA treated with omalizumab. In addition, we assessed the following secondary outcomes at baseline and after 12 months of treatment in all three study arms: (1) dependence on corticosteroid use, (2) spirometry characteristics (forced expiratory volume in 1 s FEV1), and (3) fraction of exhaled NO (FeNO). Furthermore, all treatment outcomes were analyzed in relation to the sensitization type by different aeroallergens at baseline and compared between polysensitized patients (sensitized to all tested allergens) and monosensitized or partial polysensitized patients. The main aim of the study was to test the hypothesis that different treatment outcomes may be obtained for each sensitization pattern.

## Methods

### Study design

This study was designed as a multicenter, non-interventional, observational post hoc analysis of longitudinal data of patients enrolled in the CAR in 10 specialized centers [National Centre for Severe Asthma (NCTA)] in the CR between 2007 and 2018. Data analysis had a mixed design (cross-sectional and longitudinal analyses). Data were collected at three time points: baseline and at 16 weeks and 12 months after treatment initiation.

### Study sample

From 389 patients enrolled in the CAR, 279 individuals from the 10 NCTA centers in the CR were analyzed (additional data of individual allergic sensitization were available). All patients had SAA with a proven allergy to at least one (or more) perennial airborne allergen and had ≥ 2 severe asthma exacerbations [20–22] in the year before omalizumab treatment initiation. Omalizumab treatment was maintained in 161 patients for 12 months. In 118 patients, omalizumab treatment was discontinued (in 48 patients for a lack of efficacy and in 70 patients for other reasons).

### Study procedures

The patients were evaluated by physicians before treatment initiation (baseline) and at 16 weeks and 12 months after omalizumab administration. The clinical evaluation at each visit was performed by spirometry [23] and using the FeNO analysis [24, 25]. In addition, the medical history of patients, with respect to the average dose of SCS in the 4 month before treatment (assessed and expressed as an equivalent of prednisolone), rescue medication, and severe asthma exacerbation [22], was recorded, and the patients were requested to complete an ACT questionnaire [26, 27].

At baseline, skin prick test (SPT) and laboratory assessment (total and specific IgE in international units per ml, IU/ml) were performed in all patients. A positive result in the SPT was defined as a mean wheal diameter of ≥ 3 mm [28]. All results were categorized as negative (SPT < 3 mm and/or sIgE < 0.35 IU/ml), mild positive (SPT < 10 mm and/or sIgE < 3.5 IU/ml), or strong positive (SPT ≥ 10 mm and/or sIgE ≥ 3.5 IU/ml). In cases of discordance between the SPT and specific IgE, the stronger sensitization result was considered. In the analysis of dichotomous variables, a positive result refers to any SPT and/or sIgE positivity. In our analyses, the “polysensitized” group comprised patients who were concurrently sensitized to all tested perennial allergens, that is, mites, molds, and pets (Table 1).

**Table 1:** Sensitization characteristics (per-protocol analysis)

Sensitization	Whole sample		Responders		Non-responders		Withdrew		*p*
	n	%	n	%	N	%	n	%	
n	279	100.0	161	57.7	48	17.2	70	25.1	
Monosensitization *Alternaria*	3	1.1	2	1.2	1	2.1	0	0.0	0.249
Monosensitization *Aspergillus*	12	4.3	6	3.7	1	2.1	5	7.1	
Cats and dogs	22	7.9	14	8.	4	8.3	4	5.7	
Molds, cats, and dogs	14	5.0	8	5.0	3	6.3	3	4.3	
Molds	24	8.6	12	7.5	4	8.3	8	11.4	
Mites, cats, and dogs	51	18.3	31	19.3	11	22.9	9	12.9	
Mites and molds	32	11.5	14	8.7	4	8.3	14	20.0	
Mites	70	25.1	37	23.0	16	33.3	17	24.3	
Mites, molds, cats, and dogs	51	18.3	37	23.0	4	8.3	10	14.3	
Polysensitization	51	18.3	37	23.0	4	8.3	10	14.3	0.043
Non-polysensitization	228	81.7	124	77.0	44	91.7	60	85.7	

Analysis: Chi squared test

**Fig. 1:**
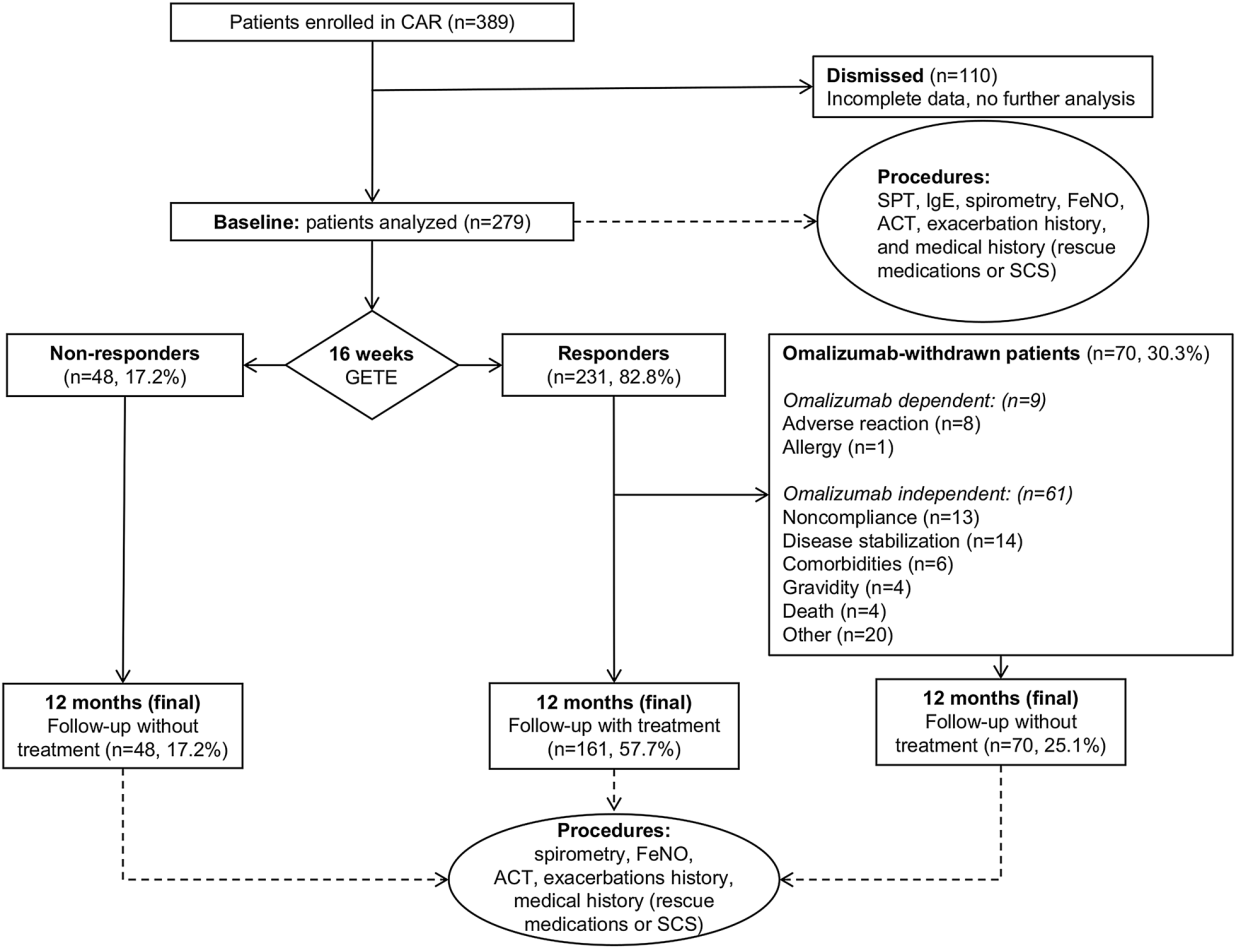
Study design. Summary of study arms and procedures

The GETE analysis was performed by physicians at 16 ± 1 weeks. The patients were rated on a five-point scale: 1, excellent (complete control of asthma); 2, good (marked improvement); 3, moderate (discernible, but limited improvement); 4, poor (no appreciable change); and 5, worsening (overall deterioration of asthma control). Patients with an “excellent” or “good” response were considered responders (n = 231) and those with a “moderate,” “poor,” or “worsening” response were considered non-responders (n = 48) [29]. Omalizumab therapy was discontinued in non-responders. Some patients who were considered as responders withdrew from therapy (n = 70) for other reasons (8 for adverse reactions, 1 for allergy to omalizumab, 13 for non-compliance, 48 for other non-omalizumab-dependent reasons). Both these groups of patients underwent another evaluation after 1 year of treatment or at follow-up. The study design is shown in Fig. 1.

### Statistical analysis

All effectiveness variables were analyzed using the per-protocol population, which comprised patients enrolled in the registry. For analyses at specific time points, all patients with available data at that time point were considered for the analysis. Descriptive statistics are expressed as mean with standard deviation (SD) [or as median with inter-quartile range (IQR) in cases of non-normal data distribution] or frequency. For comparisons of two independent samples, we used a two-sample *t* test or Mann–Whitney test in cases where the assumption of normality was not met. Large numbers of independent samples were analyzed using a one-way ANOVA or Kruskal–Wallis test in cases of non-normal data distribution. Repeated measures were treated using a general linear model for repeated measures (GLM-RM) or Wilcoxon signed-rank test in cases of normality assumption violation. In the applicable cases, the standardized effect size characteristics for GLM were reported (partial η^2^). Frequency was analyzed using the Chi squared test. Binomial dependent variables were treated using logistic regression. Results with a p value of < 0.05 were considered statistically significant. Data were analyzed using IBM SPSS Statistics for Windows, Version 22.0 (Armonk, NY: IBM Corp).

## Results

### Baseline characteristics of patients

Three hundred and eighty-nine patients with SAA treated with omalizumab as a part of a normal clinical practice were enrolled in the CAR in 10 specialized centers in the CR between 2007 and 2018. One hundred and ten patients (28.3% of all enrolled patients in the registry) were excluded from the assessment due to incomplete data in the registry. The data of 279 patients (71.7%) who completed the study were analyzed, of which, 231 (82.8%) were assigned as responders according to the GETE analysis results at 16 weeks after treatment initiation. Omalizumab treatment was further maintained only in responders. Both responders and non-responders were assessed 12 months after treatment initiation at the final point of study protocol. Seventy (30.3%) responders withdrew treatment because of various reasons, including adverse events (AEs; n = 8), signs of allergy (n = 1), and non-AE reasons (n = 61; noncompliance, disease stabilization, comorbidities, gravidity, death, administrative, and personal reasons). None of the AEs were assigned as severe. At the final study point (12 months after treatment deployment), the results of 161 (57.7%) responders, 48 (17.2%) non-responders, and 70 (25.1%) patients who withdrew were compared (Fig. 1).

The baseline characteristics of patients in the three study arms did not significantly differ in terms of sex (p = 0.488), age (p = 0.274), weight (p = 0.452), total IgE (p = 0.186), or omalizumab dose (p = 0.772). Moreover, there were non-significant differences in FeNO (p = 0.877) and FEV1 (% of predicted, p = 0.660). However, non-responders had a significantly lower baseline ACT score (median 10 points) than responders and omalizumab-withdrawn patients (median 12 points for both, p = 0.005). There were significant differences in the number of severe exacerbations during the year before treatment initiation. Both responders and non-responders experienced a median of two exacerbations, whereas omalizumab-withdrawn patients had a median of one exacerbation (p = 0.007). The proportion of patients using SCS at the time of treatment initiation also differed significantly (p = 0.015) in the three study arms: non-responders, 83.3%; responders, 73.9%; and omalizumab-withdrawn patients, 60%. Nonetheless, the doses of SCS (equivalent to prednisolone) used in the three study arms were similar (p = 0.387) (Table 2).

**Table 2:** Baseline and final characteristics (per-protocol analysis)

Characteristics	Whole sample				Responders				Non-responders				Withdrew				*p*
	Mean/n/%	SD	Median	IQR	Mean/n/%	SD	Median	IQR	Mean/n/%	SD	Median	IQR	Mean/n/%	SD	Median	IQR	
General																	
n (male/female)	279 (118/161)				161 (65/96)				48 (24/24)				70 (29/41)				0.488
Age (year)	53.79	12.48	53.00	20.00	52.96	11.35	52.00	17.00	54.06	12.18	53.50	18.50	55.51	14.96	55.00	24.00	0.274
Weight (kg)	78.42	17.40	78.00	25.00	78.24	17.36	75.00	25.00	81.52	18.19	80.00	21.00	76.69	16.91	78.50	25	0.452
Total IgE (IU/ml)	355.47	325.88	244.00	366.00	349.52	311.55	225.00	386.00	399.44	317.69	319.50	352.00	339.00	363.78	207.50	361.00	0.186
Omalizumab dose (mg/month)	472.04	250.36	450.00	300.00	469.10	246.94	300.00	300.00	509.38	299.58	450.00	375.00	453.21	220.59	300.00	300.00	0.772
Baseline																	
FeNO (ppb)	51.38	46.31	38.00	52.00	51.01	48.52	38.00	51.00	46.80	33.53	38.00	50.00	55.81	48.76	44.00	56.00	0.877
ACT (points)	12.34	4.10	12.00	6.00	12.55	3.86	12.00	6.00	10.64	4.04	10.00	5.00	13.03	4.42	12.00	7.00	*0.005*
FEV1 (%pred)	64.45	18.17	66.00	24.00	63.58	18.20	65.00	24.00	65.81	16.80	66.50	22.50	65.51	19.11	67.50	19.00	0.660
Severe exacerbations (n/year)	2.55	3.80	2.00	2.00	2.78	4.55	2.00	2.00	2.88	2.51	2.00	2.00	1.80	2.20	1.00	2.00	*0.007*
Systemic corticosteroids (n/%)	201/72,0%				119/73,9%				40/83,3%				42/60,0%				*0.015*
Prednisone (mg/day)	13.84	14.19	10.00	15.00	13.76	16.03	8.00	15.00	15.38	14.17	10.00	15.00	12.62	6.99	10.00	11.00	0.387
Final																	
FeNO (ppb)	40.82	46.97	26.00	29.00	35.19	36.62	24.00	27.00	43.61	40.70	34.00	31.00	52.72	67.56	32.00	38.00	0.158
ACT (points)	17.45	5.14	18.00	8.00	18.36	4.74	19.00	7.00	13.45	5.22	12.50	9.00	18.03	4.78	18.00	8.00	*< 0.001*
FEV1 (%pred)	72.14	21.90	73.00	29.00	72.16	22.12	71.00	29.00	69.23	17.96	66.00	24.00	74.06	23.81	76.50	31.00	0.278
Severe exacerbations (n/year)	1.03	2.64	0.00	1.00	0.94	2.59	0.00	1.00	1.81	3.88	1.00	2.00	0.70	1.37	0.00	1.00	*0.022*
Systemic corticosteroids (n/%)	121/43,4%				68/42,2%				27/56,3%				26/37,1%				0.109
Prednisone (mg/day)	10.40	12.63	5.00	6.00	9.07	11.41	5.00	18.00	16.78	18.06	10.00	15.00	7.23	4.19	8.50	5.00	*0.008*

Analysis: Kruskal–Wallis test, Chi-square test

### Time-dependent changes

**Fig. 2:**
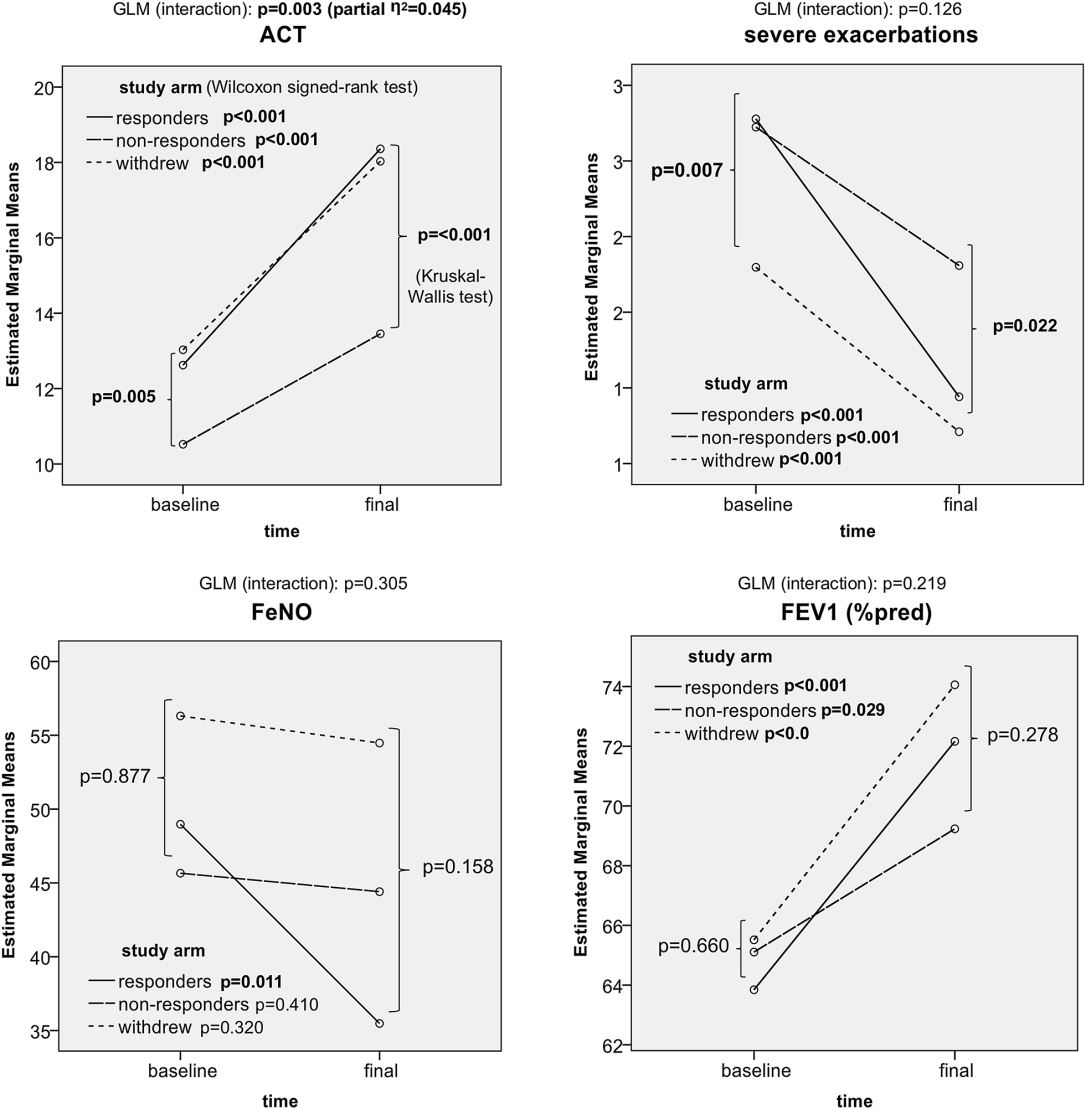
Clinical outcomes in responders, non-responders, and omalizumab-withdrawn patients. Time-dependent changes in ACT, severe exacerbations, FeNO, and FEV1 in relation to study arm (analysis: GLM, Wilcoxon signed-rank test)

All measured parameters (ACT, annual rate of severe exacerbations, FeNO, FEV1, and SCS dose) improved during the 1-year treatment period in responders. In addition, a significant improvement was observed in non-responders and omalizumab-withdrawn patients (excluding FeNO). The analysis of mutual interaction between the time factor and study arm factor presented significant results only for ACT score changes (p = 0.003, partial η^2^ = 0.045) and SCS dose reduction (p = 0.045, partial η^2^ = 0.031), with a rather low effect size (Fig. 2 and Table 2).

**Fig. 3:**
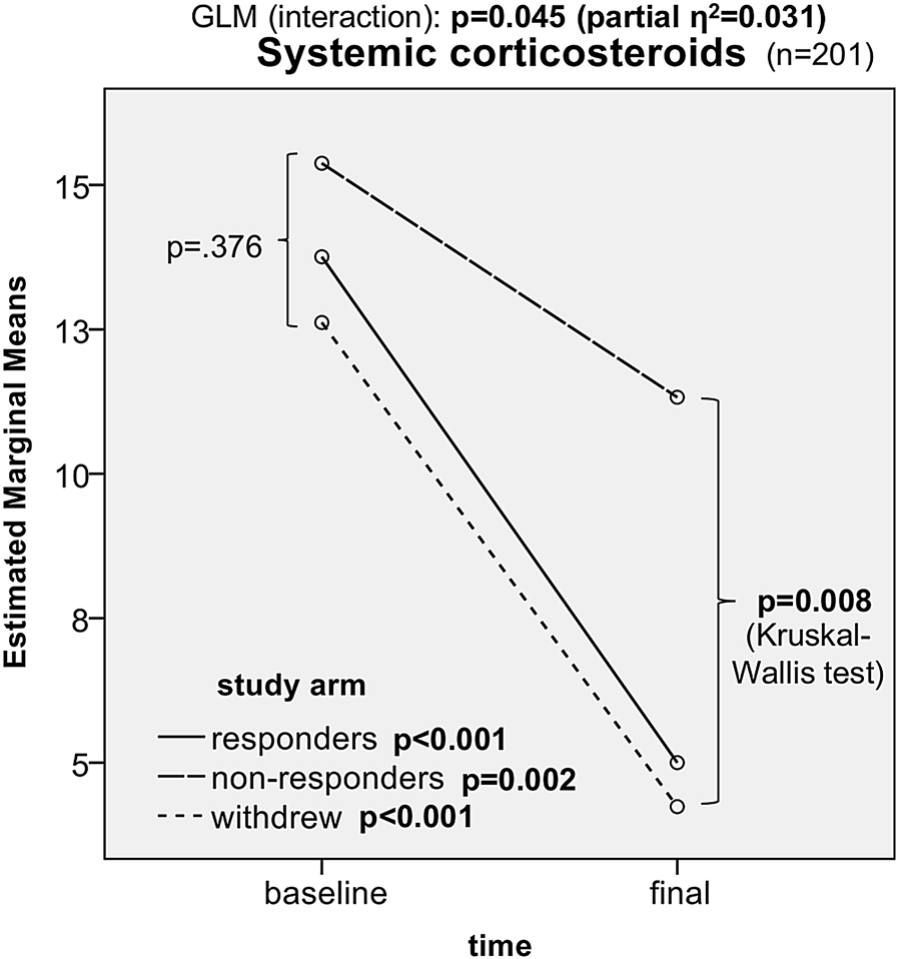
Tapering of systemic corticosteroids in responders, non-responders, and omalizumab-withdrawn patients. Time-dependent changes in systemic corticosteroid doses (analysis: GLM, Wilcoxon signed-rank test)

**Fig. 4:**
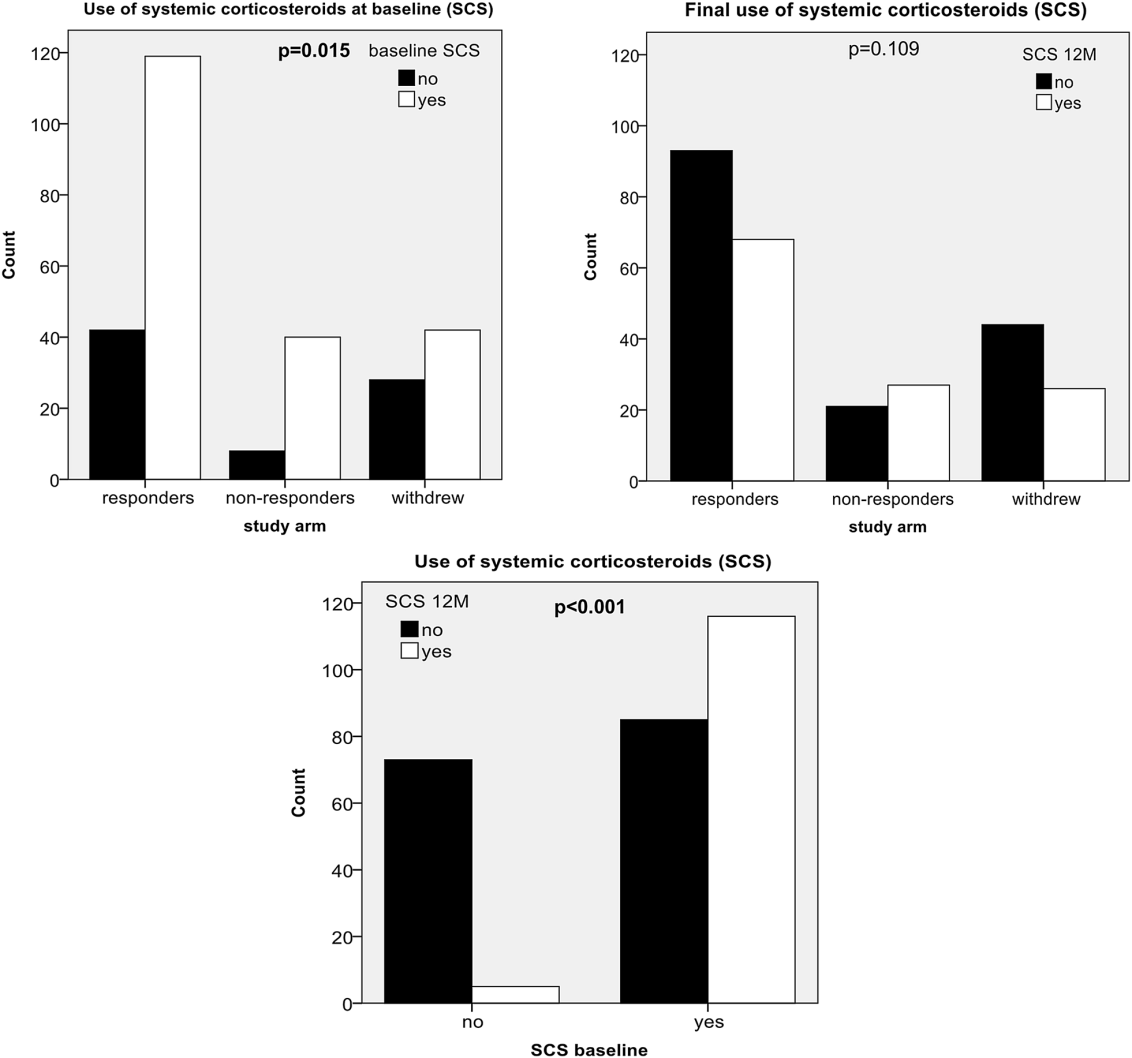
Baseline and final use of corticosteroids in responders, non-responders, and omalizumab-withdrawn patients. Baseline patient count of systemic corticosteroid use and counts of systemic corticosteroid use in each study arm

We also observed a substantial decrease in the proportion of patients using SCS in all the study arms. The most significant decrease was observed in the responder group (from 73.9% to 42.2%); 42.3% of patients using SCS at baseline were allowed to discontinue the drugs, and only five patients initiated SCS during the study period de novo (p < 0.001) (Figs. 3 and 4; Table 2).

### Sensitization characteristics

**Fig. 5:**
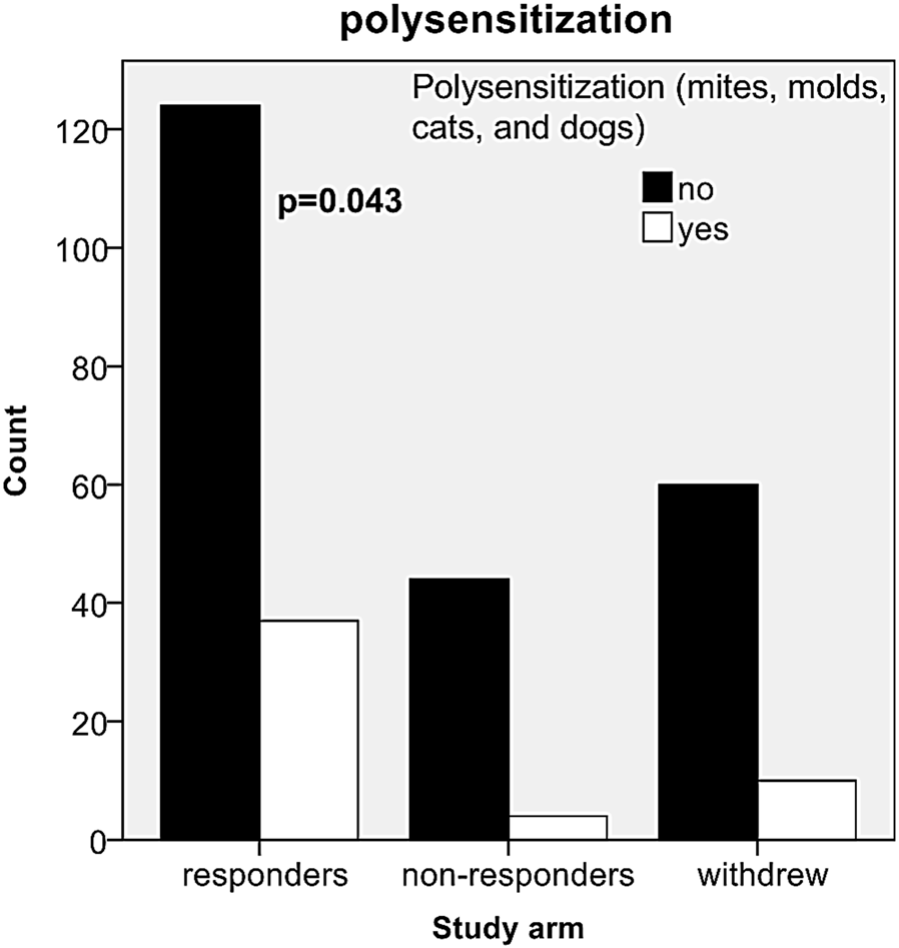
Frequency of polysensitization in responders, non-responders, and omalizumab-withdrawn patients. Counts of patients with polysensitization in relation to the study arm

We found no significant differences in the ratio of sensitization to particular perennial allergens or allergen groups among all study arms [assessed either semi-quantitatively or dichotomously, p = 0.249, except for a concurrent sensitization to all tested perennial allergens (i.e., mites (*Dermatophagoides pteronyssinus* and/or *D. farinae*), molds (mixture of molds and/or *Aspergillus* spp. and/or *Alternaria* spp.), cats, and dogs)], and they were assigned to a polysensitized subgroup of patients. The highest proportion (72.5%) of all polysensitized subjects was recruited from the responders arm (23% of responders, p = 0.043) (Fig. 5 and Table 1).

**Fig. 6:**
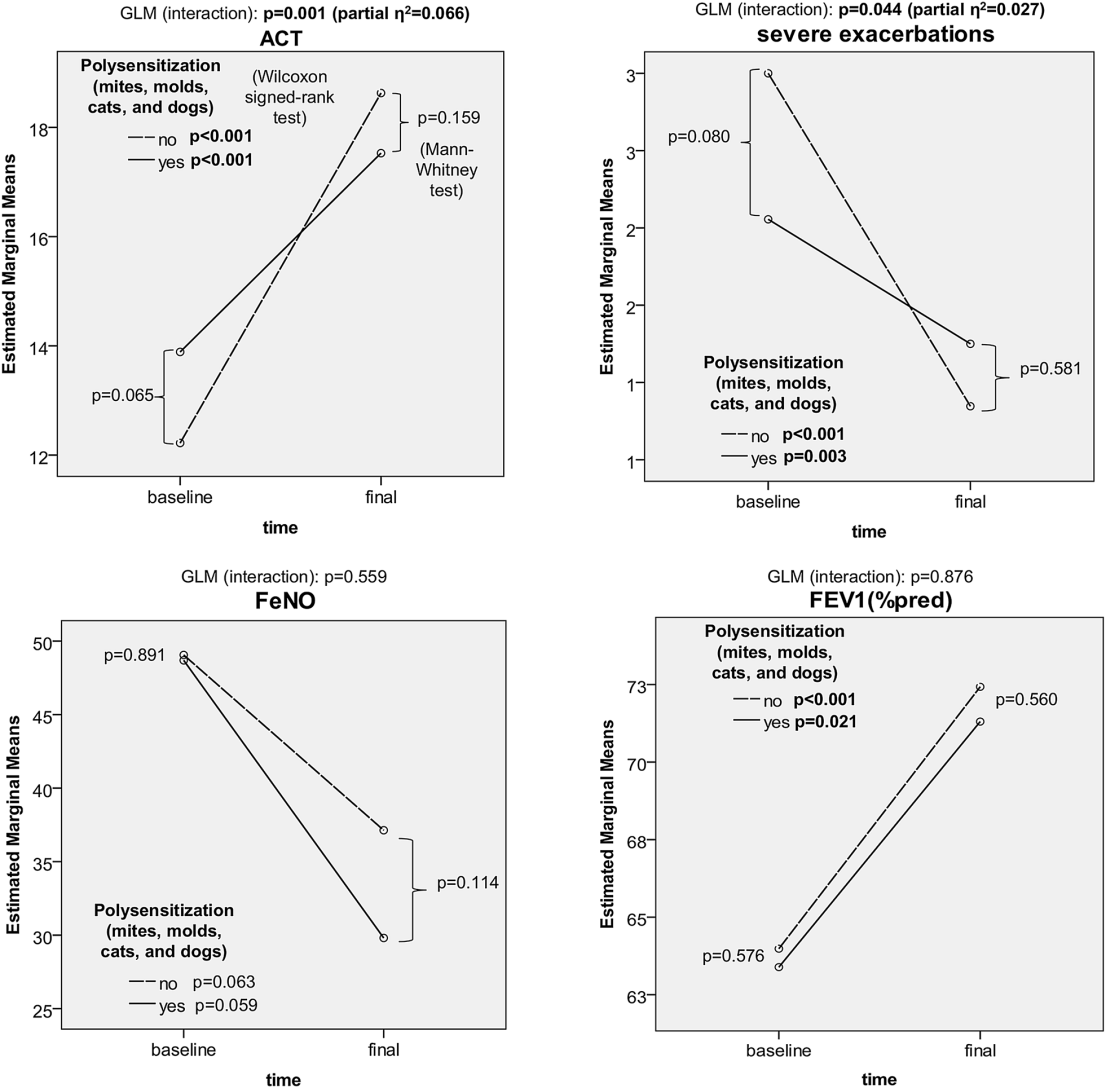
Clinical outcomes in polysensitized and non-polysensitized patients. Time-dependent changes in ACT, severe exacerbations, FeNO, and FEV1 in relation to polysensitization at baseline (analysis: GLM, Wilcoxon signed-rank test)

**Fig. 7:**
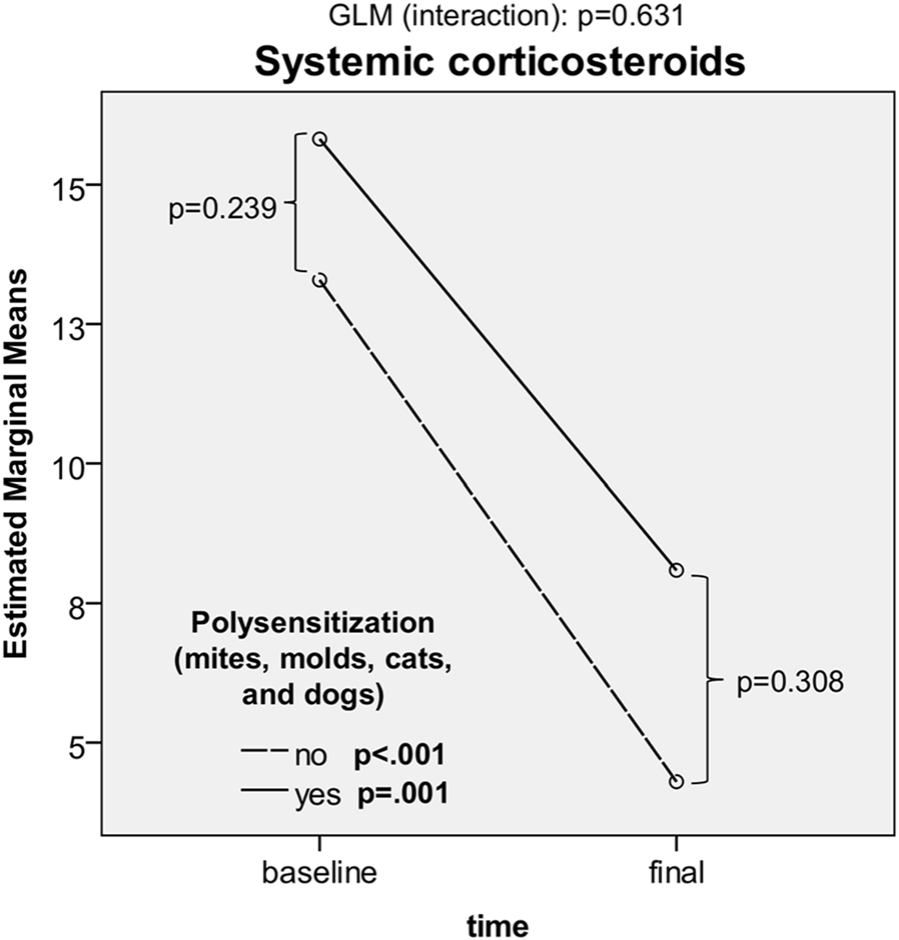
Tapering of systemic corticosteroids in polysensitized and non-polysensitized patients. Time-dependent changes in systemic corticosteroid doses (analysis: GLM, Kruskal–Wallis test, Wilcoxon signed-rank test)

**Fig. 8:**
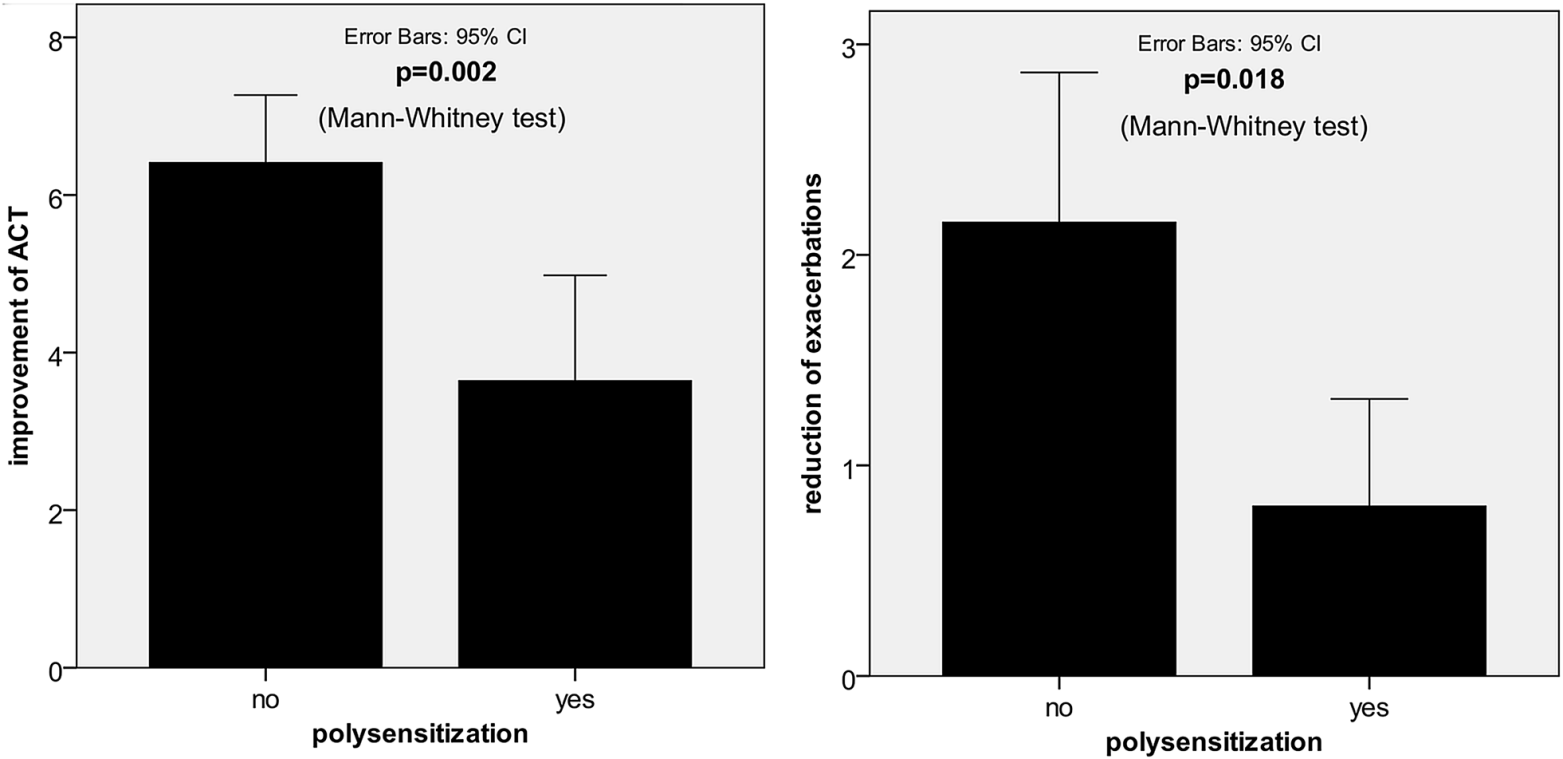
Asthma control test (ACT) result improvement and reduction of annual number of severe exacerbations in polysensitized and non-polysensitized patients (analysis: Mann–Whitney test)

No significant difference was observed in age, weight, total IgE, or omalizumab dose between the polysensitized and non-polysensitized responders (Table 3). The clinical characteristics of polysensitized patients were independent of wheal diameter or specific IgE levels to particular allergens. However, we observed some clinically relevant differences. Polysensitized patients in the responders study arm exhibited a lower tendency to improve the ACT result (a mean improvement of 6.41 points in non-polysensitized vs. 3.64 points in polysensitized subgroup, p = 0.002) and a lower tendency to reduce the annual number of exacerbations (a mean reduction of 2.15 in non-polysensitized subgroup vs. 0.81 in polysensitized subgroup, p = 0.018) compared with non-polysensitized patients (Fig. 8). This difference led to significant interactions between polysensitization and time-dependent changes in ACT and exacerbation rate (ER) reduction in the GLM-RM analysis (p = 0.001, partial η^2^ = 0.066 for ATC improvement and p = 0.044, partial η^2^ = 0.027 for ER reduction) despite non-significant differences between these two populations at particular study points (Figs. 6 and 7).

**Table 3:** Polysensitization

Characteristic	No polysensitization				Polysensitization				*p*
	Mean/n/%	SD	Median	IQR	Mean/n/%	SD	Median	IQR	
General									
n (male/female)	124 (50/74)				37 (15/22)				0.981
Age (years)	53.36	10.94	52.00	17.00	51.62	12.68	52.00	18.00	0.546
Weight (kg)	77.64	17.23	75.00	24.50	80.27	17.88	80.00	27.00	0.385
Total IgE (IU/ml)	328.83	294.71	204.50	377.00	418.84	358.10	304.00	444.00	0.178
Omalizumab dose (mg/month)	483.87	247.80	450.00	300.00	419.59	240.74	300.00	300.00	0.109
Baseline									
FeNO (ppb)	51.26	50.23	38.00	48.00	50.14	42.93	38.00	59.00	0.891
ACT (points)	12.19	3.68	12.00	5.50	13.73	4.23	14.00	7.00	0.065
FEV1 (%pred)	63.61	18.65	66.50	24.00	63.49	16.83	61.00	15.00	0.576
Severe exacerbations (n/year)	2.98	4.89	2.00	3.00	2.08	3.14	1.00	1.00	0.080
Systemic corticosteroids (n/%)	97 (78.2%)				22 (59.5%)				0.023
Prednisone (mg/day)	13.29	14.86	7.00	15.00	15.82	20.71	10.00	15.00	0.239
Final									
FeNO (ppb)	37.01	37.66	24.00	26.00	28.78	32.50	20.00	32.00	0.114
ACT (points)	18.63	4.81	20.00	7.00	17.53	4.48	17.00	7.50	0.159
FEV1 (%pred)	72.43	23.14	76.00	30.00	71.31	18.71	68.50	22.50	0.560
Severe exacerbations (n/year)	0.85	2.46	0.00	1.00	1.25	3.01	0.00	1.00	0.581
Systemic corticosteroids (n/%)	54 (43.5%)				14 (20.6%)				0.537
Prednisone (mg/day)	7.94	8.95	5.00	8.00	13.43	17.87	5.00	5.00	0.308

Baseline and final characteristics of responders with regards to polysensitization (per protocol analysis, Wilcoxon signed-rank test, and Chi squared test)

### Logistic regression

**Fig. 9:**
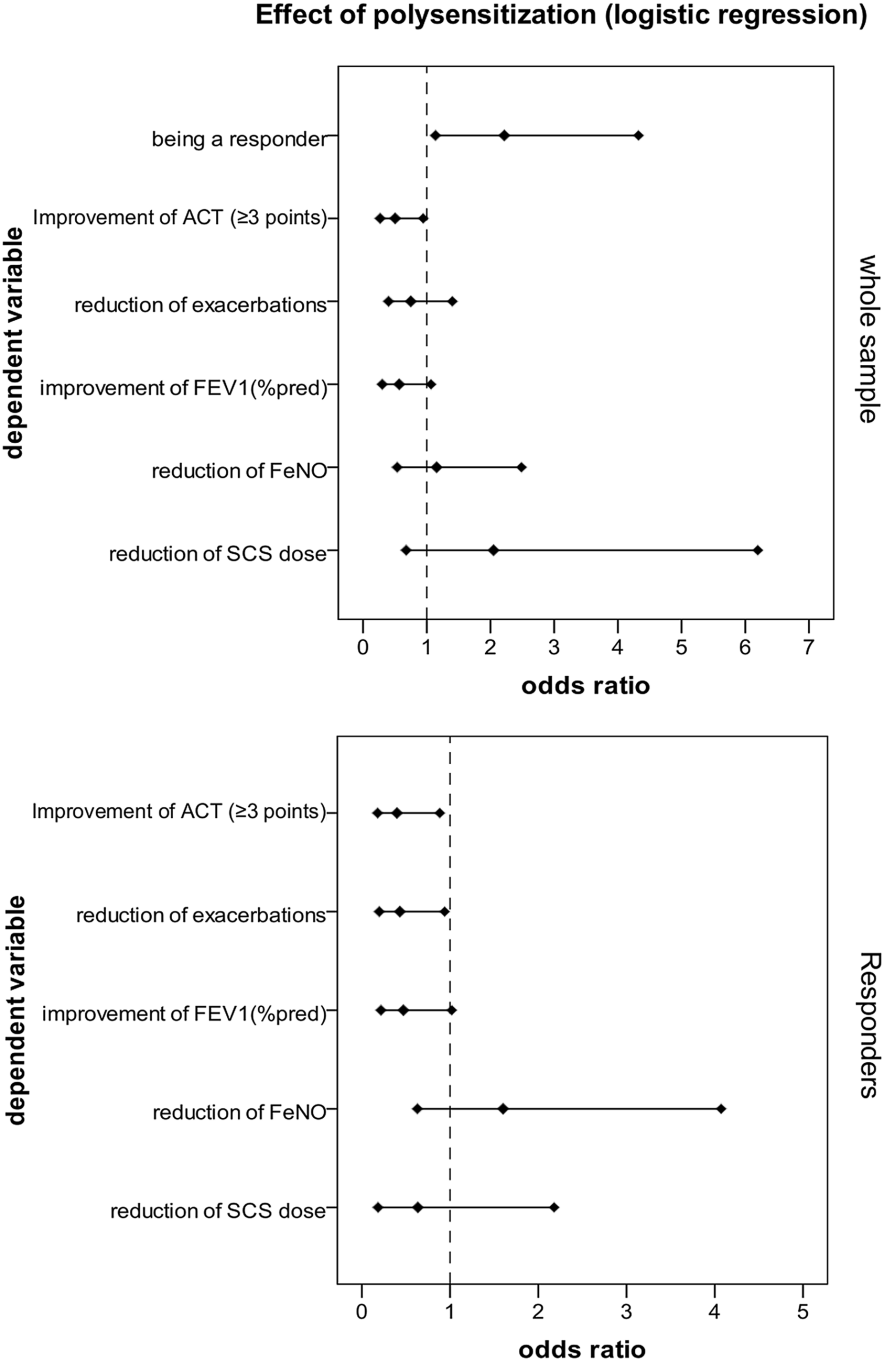
Forest plot and odds ratio of selected variables in relation to polysensitization. OR of the selected outcome measures in relation to polysensitization at baseline (analysis: binary logistic regression)

We performed a meticulous logistic regression analysis of the influence of polysensitization on the odds of being a responder regarding a clinically significant improvement in the ACT result (i.e., ≥ 3 points), a reduction in exacerbations (Fig. 8), an improvement in FEV_1_ (% of predicted), a reduction in FeNO, and a reduction in SCS dose. A significant positive effect of polysensitization on odds of being a responder (OR = 2.217, p = 0.02) and negative effect on odds of ACT improvement (OR = 0.503, p = 0.032) were observed in the whole sample analysis. In other words, polysensitized patients exhibited a higher tendency to be a responder, but a lower tendency to increase ACT ≥ 3 points than non-polysensitized patients (Fig. 9, Table 4).

**Table 4:** Binary logistics regression (effect of polysensitization)

Sample	n	Dependent variable	OR	95% CI for OR		*p*
				Lower	Upper	
Whole sample	279	Being a responder	2.217	1.137	4.323	0.02
Whole sample	279	Improvement in ACT (≥ 3 points)	0.503	0.268	0.944	0.032
		Reduction in exacerbations	0.749	0.4	1.401	0.365
		Improvement in FEV1(%pred)	0.567	0.301	1.066	0.078
		Reduction in FeNO	1.155	0.536	2.486	0.713
All patients receiving SCS	201	Reduction in SCS dose	2.048	0.677	6.196	0.205
Responders	161	Improvement in ACT (≥ 3 points)	0.398	0.179	0.883	0.023
		Reduction of exacerbations	0.431	0.198	0.938	0.034
		Improvement in FEV1(%pred)	0.471	0.217	1.019	0.056
		Reduction in FeNO	1.601	0.63	4.072	0.323
All responders receiving SCS	119	Reduction in SCS dose	0.635	0.184	2.179	0.474

Forest plot and OR with regards to poly sensitization

We obtained similar results in the sub-analysis of responders. Polysensitized responders revealed a lower tendency to improve the ACT result (OR = 0.398, p = 0.023) and reduce ER (OR = 0.431, p = 0.034) than non-polysensitized patients.

## Discussion

In the present study, we evaluated the effect of sensitization to individual allergens or their combinations on the outcomes of anti-IgE therapy in patients with SAA. Polysensitized patients showed a higher tendency to be a responder than non-polysensitized patients, and a lower tendency to increase ACT and reduce ER.

There is strong evidence to indicate that anti-IgE therapy has an impressive immuno-modulatory effect [30, 31] and broad clinical effects [31–33], notably a decrease in the daily dose of SCS or even its termination and an improvement in the quality of life, as documented in several real-setting studies [14]. There are also rare reports of the suppression of allergic reactivity [34–37] or susceptibility to viral infections by anti-IgE therapy [38]. Other studies on the clinical characteristics of patients have shown optimal clinical benefits of the treatment. The biomarker-based prospective study EXTRA demonstrated that omalizumab treatment tends to be more effective in patients with elevated baseline eosinophil count, FeNO, and serum periostin level [39]. However, these results have not been confirmed in the retrospective real-life analysis of omalizumab-treated patients, the STELLAIR study, which showed a clinically significant effect irrespective of baseline eosinophil count [40]. Similar results have been reported by the prospective observational study PROSPERO, which reported that the effects of omalizumab (a reduction in ER and number of hospitalizations, and an improvement in ACT) were independent of either baseline eosinophil count or FeNO [41]. We should be aware of instability of peripheral blood immune parameters over a year in patients with stable asthma [42].

Notably, evidence regarding the influence of sensitization profile on omalizumab treatment outcomes is scarce. Currently, it is broadly accepted that the frequency of sensitization in a population to a particular allergen depends on the climate or environment; different biological features of causal allergens can determine the different clinical traits of allergy [43]. However, there are only a few studies on the clinical characteristics of patients with asthma related to the sensitization profile. A previous study in a Chinese population demonstrated that sensitization to house dust mites was associated with increased severity of asthma [44]. In contrast, another study (in Spain) reported that sensitization to different allergens was not associated with significant differences in severity and control of asthma. However, diagnostic and therapeutic approaches slightly differ according to individual allergen sensitization [45]. Moreover, there was no mention of anti-IgE treatment.

In the CR, patients with severe uncontrolled asthma are treated in specialized sites of NCTA [46, 47]. Only these centers are eligible to indicate biological treatment with anti-IgE antibody (omalizumab) in the CR [7, 47]. This careful approach may contribute to the higher number of responders (according to GETE) in the eXpeRience registry (88.9%) [15] than in data from other countries (69.9%) [48]. Extensive supervision of patients in the NCTA centers can result in better treatment outcomes with respect to some parameters in all groups of patients.

In our analysis of patients, whose data are included in the CAR registry, we reported similar response rates according to the GETE analysis at 16 weeks after treatment initiation (82.8%). However, the treatment outcomes were remarkable in all groups of patients (including non-responders and omalizumab-withdrawn patients). This might be due to the fact that all patients were treated with omalizumab for at least 16 weeks (non-responders), 12 months (responders), and between 16 weeks and 12 months (omalizumab-withdrawn patients). Another reason could be the unequal number of patients in all study arms. In addition, there may be a bias in results of responders compared with that of omalizumab-withdrawn patients, because all patients in the withdrawn group were a subgroup of responders. Moreover, 14 patients (20%) in this group discontinued treatment because of the stabilization of disease. Other reasons for withdrawal were diverse, but none of them was worsening of the disease. Although Namazy et al. [49, 50] proved that the use of omalizumab in pregnant women was safe, we preferred to terminate treatment by a mutual agreement with pregnant women in some centers. The outcomes were not influenced by the fact that non-responders appeared to have more severe disease than other patients (Table 2).

Lombardi et al. [43] reported that some aeroallergens, especially molds, pet dander, cockroach, and ragweed, were found to be more strongly associated with severe asthma. The association between mold sensitization and severe asthma is well known and conceptualized as severe asthma associated with fungal sensitization [51]. The identification of related causal allergen(s) is important for optimal complex therapeutic strategies, including specific allergen immunotherapy [52]; however, evidence of allergen avoidance is under debate [53].

In our study, we assessed mites instead of cockroaches because the representation of patients with sensitization to cockroach in the Czech population is weak compared with that in other countries (e.g., the USA). In the subgroup of monosensitized or polysensitized patients, there was no significant difference in treatment outcomes regarding GETE (Table 1) and no difference in asthma severity. Nevertheless, polysensitized patients (as defined above) had higher odds (OR = 2.217, p = 0.02) of being responders than all other subgroups of non-polysensitized patients. In contrast, they had reduced odds of ACT improvement (OR = 0.503, p = 0.032) among all other subgroups (OR = 0.398, p = 0.023 in the responder subgroup) and a reduction in exacerbation (OR = 0.431, p = 0.034 in the responder subgroup) compared with the non-polysensitized patients. This can be explained by the tendency (although nonsignificant) of polysensitized patients to have a higher baseline ACT score and lower rate of severe exacerbations than non-polysensitized patients. In addition, these patients tend to have a better clinical status at treatment initiation than non-polysensitized patients (Fig. 8). We suggest that these polysensitized patients with asthma may have a different subgroup of allergic diseases that may share some features with the “Th2-ultrahigh” concept suggested by Peters [54]. However, we do not have enough data to confirm this possible connection.

There were certain limitations to our study. First is the low number of patients with a distinct sensitization profile, which led to a loss of statistical power. However, a substantial number of patients from the initial pool (n = 389) had to be excluded from the assessment (n = 110, 28.3% of all enrolled patients in the registry) due to incomplete data in the registry. The used per-protocol analysis was not designed to treat censored (or missing) data. Second, we lost some information owing to the semiquantitative evaluation of categorized levels of sensitization. Third, we could not assess differences in treatment outcomes among patients sensitized to perennial or seasonal allergens (including weeds) because virtually all patients were concurrently sensitized to at least one (or mostly more) seasonal allergen and at least one (or more) perennial allergen, which was a necessary pre-condition for treatment initiation according to the omalizumab Summary of Product Characteristics. Fourth, there are some conflicting results regarding baseline ACT, an ER in responders; this could be caused only by sampling error. However, due to the exploratory nature of the study, we believe that it is important to remark all interesting data configuration to highlight potential variables for future confirmatory studies. Thus, we are unable to suggest a simple and clear clue to deal with the kind of sensitisation of patients with asthma receiving omalizumab therapy. We rather focused on seeking potential variables and parameters that could be used as putative biomarkers to estimate the supposed disease evolution and possible treatment outcomes. Finally, because of the observational nature of the study, we did not exploit randomization and placebo control group.

## Conclusions

In summary, this is the first study to provide data on the effects of allergen sensitization of patients with allergic asthma on omalizumab treatment response. We believe that there may be some biological differences among distinct subpopulations of patients with asthma in terms of polyvalence of atopic sensitization. These differences may lead to distinct objective treatment effects (e.g., reduction in severe exacerbations) and different subjective disease perception (e.g., improvement in the ACT result).

We found that polysensitized patients exhibited a higher tendency to be responders than non-polysensitized patients but a lower tendency of showing an improvement in the ACT results and a reduction in ER. We suggest that these polysensitized patients with asthma may constitute a separate subgroup of individuals with different allergic diseases and distinct clinical characteristics. Prospective data from controlled trials are needed to confirm these observations.

## Data Availability

Not applicable.

## Abbreviations

ACT: Asthma control test
ER: Exacerbation rate
CAR: Czech Anti-IgE Registry
GETE: Global evaluation of treatment effectiveness
SCS: Systemic corticosteroid
SAA: Severe allergic asthma
NCTA: National Centre for Severe Asthma
FEV1: Forced expiratory volume in 1s
FeNO: Fraction of exhaled NO
